# Antidiabetic Effect of *Morinda citrifolia* (Noni) Fermented by *Cheonggukjang* in KK-A^**y**^ Diabetic Mice

**DOI:** 10.1155/2012/163280

**Published:** 2012-08-27

**Authors:** So-Young Lee, So-Lim Park, Jin-Taek Hwang, Sung-Hun Yi, Young-Do Nam, Seong-Il Lim

**Affiliations:** Fermentation and Functionality Research Group, Korea Food Research Institute, Gyeonggi-Do, Sungnam-Si 463-746, Republic of Korea

## Abstract

Antidiabetic effects of *Morinda citrifolia* (aka Noni) fermented by *Cheonggukjang* (fast-fermented soybean paste) were evaluated using a T2DM (type 2 diabetes mellitus) murine model. Six-week-old KK-Ay/TaJcl mice were randomly divided into four groups: (1) the diabetic control (DC) group, provided with a normal mouse diet; (2) the positive control (PC) group, provided with a functional health food diet; (3) the *M. citrifolia* (MC) group, provided with an MC-based diet; (4) the fermented *M. citrifolia* (FMC) group, provided with an FMC-based diet. Over a testing period of 90 days, food and water intake decreased significantly in the FMC and PC groups compared with the DC group. Blood glucose levels in the FMC group were 211.60–252.20 mg/dL after 90 days, while those in the control group were over 400 mg/dL after 20 days. In addition, FMC supplementation reduced glycosylated hemoglobin (HbA1c) levels, enhanced insulin sensitivity, and significantly decreased serum triglycerides and low-density lipoprotein (LDL) cholesterol. Furthermore, a fermented *M. citrifolia* 70% ethanolic extract (FMCE) activated peroxisome proliferator-activated receptor-(PPAR-) **γ** and stimulated glucose uptake via stimulation of AMP-activated protein kinase (AMPK) in cultured C2C12 cells. These results suggest that FMC can be employed as a functional health food for T2DM management.

## 1. Introduction

Diabetes mellitus (DM) is a chronic disorder characterized by hyperglycemia, with disturbances in carbohydrate, lipid, and lipoprotein metabolism stemming from defects in insulin secretion and insulin action, or both. About 90–95% of all cases of diabetes mellitus can be attributed to type 2 diabetes mellitus (T2DM, or adult-onset diabetes). T2DM is caused by the nonresponsiveness of cells to insulin rather than by defective insulin production. If not treated, T2DM can result in numerous severe complications, including coronary artery disease, peripheral vascular disease, and even death due to hyperglycemia [1]. T2DM currently affects more than 246 million people worldwide, and it is estimated that the number of patients with diabetes mellitus will increase to 360 million by 2030 [2]. Indeed, about 5–10% of the total health care budget has been used for treatment and management of T2DM in many countries [3]. 

 Management of T2DM usually begins with a change in diet and exercise. Nonetheless, most patients ultimately require pharmacotherapy, such as injected insulin and/or oral antidiabetic drugs. Many oral antidiabetic drugs are available for the treatment and control of the symptoms of T2DM, such as sulfonylurea agents, biguanides (e.g., metformin), thiazolidinedione (TZD) drugs (also known as glitazones), *α*-glucosidase inhibitors, and glucagon-like peptide-1 (GLP-1) inhibitors. However, these drugs can cause serious adverse effects, among them hypoglycemia, hepatic toxicity, weight increase, physconia (enlargement of the abdomen), and lactic acidosis [4]. Thus, there is an increasing demand for natural products (and in particular, nutraceuticals) that have fewer adverse effects and higher antidiabetic efficacy. Many studies have been conducted on natural products that efficiently and safely reduce blood glucose levels in diabetic patients, and several diabetes mellitus hundred plants have reported beneficial effects. For example, *Lagerstroemia speciosa *[5], *Gymnem sylcestre *[6], *Mori folium *[7], and *Psidiium guajava *[8] are currently sold commercially as complementary and alternative medicinal materials for the treatment of diabetes. 

*Morinda citrifolia*, also called Noni, is a tropical plant that grows widely throughout the Pacific*. M. citrifola *has traditionally been used for the prevention, and treatment of arthritis, circulatory weakness, diabetes, cancer, and skin inflammation [9–12], and is widely known as a health food and a dietary supplement. A growing number of pharmacological studies on *M. citrifolia* have been published in recent years, but many of the reports are only accessible as congress abstracts. In addition, few peer-reviewed research manuscripts are available that discuss the antidiabetic activity of *M. citrifolia*, even though *M. citrifolia* has been customarily used to treat diabetes. In fact, only a single study has presented evidence, limited at best, for the antidiabetic effect of *M. citrifolia* fruit juice in an animal model of T2DM [13]. The present study therefore focused on the antidiabetic properties of *M. citrifolia* and fermented* M. citrifolia* (FMC) in KK-Ay obese/diabetic mice, which are afflicted with a genetically induced form of diabetes that manifests itself as hyperinsulinemia, a result of insulin resistance [14]. *M. citrifolia *was fermented using *Cheonggukjang*, a traditional fast-fermented Korean soybean paste that was itself fermented using various *Bacillus* spp. After demonstrating the efficacy of FMC, we then aimed to elucidate the antidiabetic mechanisms of FMC. 

## 2. Materials and Methods

### 2.1. Preparation of FMC

**Figure 1:**
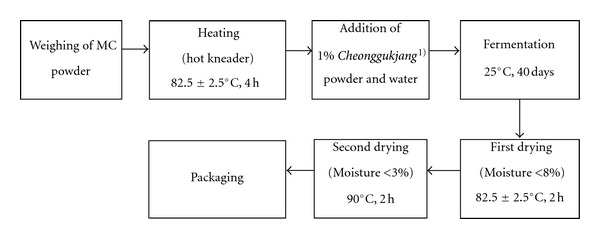
Process of fermentation. MC powder was first weighed and heated. Next, *Cheonggukjang* was added to initiate fermentation. *Cheonggukjang* is a type of fast-fermented soybean paste that is made by cooking the legumes until they are tender and then incubating them with diverse *Bacillus* species in a warm place. In this study, the dominant *Bacillus* microbes were *Bacillus* sp. (KCTC 11351BP), *Bacillus subtilis *(KCTC 11352BP), *Bacillus sonolensis *(KCTC 11354BP), and *Bacillus circulans *(KCTC 11355BP).

Fifteen kilogram of dried *M. citrifolia *(MC) fruitpowder (Bobsaewoo Seoul, Korea) was heated in a hot kaystion (300–500 L, Daehan Food Machine, Inc., Gimpo, South Korea) at 82.5 ± 2.5°C for 4 h. *Cheonggukjang* (1%, employed to initiate fermentation) containing soybeans, *Bacillus* sp. (KCTC 11351BP), *Bacillus subtilis *(KCTC 11352BP), *Bacillus sonolensis *(KCTC 11354BP), *Bacillus circulans *(KCTC 11355BP), and water (40% w/w) were added to the MC powder. For ripening, the mixture was moved to barrels (Japanese cedar, 10 L (40 × 35 cm)) that contained nine holes for ventilation. The powder was then fermented at 25°C for 40 days. Fermented MC (FMC) powder was dried at 82.5 ± 2.5°C for 2 h, and then dried at 90°C for another 2 h to decrease the moisture content of the final product to <3% and to inactivate microorganisms related with fermentation. The flow diagram for this process is shown in Figure 1. Dried FMC was packaged and stored at −20°C prior to experimentation. 

### 2.2. Extraction of FMC

The dried and milled MC and FMC were added to water or 70% ethanol (10X the sample amount w/v) with stirring for 24 h at 25°C. The extracts were centrifuged for 10 min at 3000 rpm, and the supernatants were filtered through Whatman no. 5 filter paper. The filtrates were evaporated by a rotary evaporator (RE200; Yamato Co., Tokyo, Japan) under reduced pressure at a temperature lower than 40°C. Next, the resulting concentrates were freeze-dried and storedat −20°C prior to experimentation.

### 2.3. Animals, Diets, and Treatments

Twenty-eight KK-Ay/TaJcl mice (5 weeks old, male) with genetic obese type II diabetes were purchased from Clea Japan Inc. (Tokyo, Japan). After acclimating for 1 week, the mice were divided into four groups of seven animals each, as follows: diabetic control animals (DC group), banaba-fed animals (positive control (PC) group), MC-fed animals (MC group), and FMC-fed animals (FMC group). Banaba (*Lagerstroemia speciosa*) leaves are commercially sold as a functional health food intended for the regulation of blood glucose levels. Diabetic control animals were freely fed on a normal mouse diet (AIN-93G food). PC, MC, and FMC groups were freely fed on AIN-93G blended with freeze-dried banaba, MC, or FMC powder (0.4%). All mice were maintained in the same room under conventional conditions with a regular 12 h light/dark cycle and temperature and relative humidity maintained at 23 ± 2°C and 50 ± 5%, respectively. At the end of the experimental period (90 days), the animals were fasted overnight, anaesthetized with an intraperitoneal injection of zoletil 50 (30 mg/kg), and sacrificed for the procurement of tissue samples. The study was approved by the Ethics Committee of the Experimental Animal Research Laboratory, Korea Food Research Institute (approval code, KFRI-M-10020).

### 2.4. Analysis of Blood Glucose Levels

Whole blood was sampled from the tail vein of mice without fasting at a fixed time every 10 days over a period of 90 days, and the blood sample was analyzed using the glucose oxidase method, which utilizes a blood glucose sensor strip (ACCU-CHEK Sensor, Roche, Basel, Switzerland). 

### 2.5. Analysis of Glycosylated Hemoglobin (HbA1c) Levels

At the end of the 90-day experimental period, mice were subjected to fasting overnight. Next, a fasting whole blood sample (4 *μ*L) was obtained via retro-orbital sinus puncture. The blood sample was injected into an A1c test cartridge and then analyzed by using a Hemoglobin A1c Testing Analyzer (Asan Pharmaceutical, Seoul, Korea). 

### 2.6. Serum Analysis

After 90 days of dietary treatment, KK-Ay/TaJcl mice were sacrificed, and fasting blood samples were collected and centrifuged at 3000 rpm for 10 min to obtain serum samples for serum chemical analysis. Triglyceride, total cholesterol, high-density lipoprotein (HDL)-cholesterol, and low-density lipoprotein (LDL) cholesterol were measured by an ADVIA Automated Hematology Analyzer (ADVIA 1650, Bayer, USA). Fasting serum glucose levels were assessed using a glucometer, which employed the glucose oxidase/peroxidase reaction. This reaction allows the colorimetric detection of H_2_O_2_ for the estimation of free glucose in the serum. Fasting serum insulin levels were measured with a murine insulin TMB (tetramethyl benzidine) enzyme-linked immunosorbent assay (ELISA) kit (Shibayagi, Gunma, Japan). A homeostasis model assessment of insulin resistance (HOMA-IR), an index of insulin resistance, was calculated using fasting insulin and glucose concentrations according to the following equation:

(1) HOMA-IR =(fasting  insulin  (μIU/mL)×fasting  glucose  (nmol/L))22.5.

### 2.7. Muscle Differentiation and Glucose Uptake Assay

C2C12 cells were grown in Dulbecco's modified eagle medium (DMEM) supplemented with 10% fetal bovine serum (FBS), penicillin (120 unit/mL), and streptomycin (75 *μ*g/mL) in a 5% CO_2_ atmosphere. Differentiation medium (DMEM medium with 1% horse serum) was added to confluent C2C12 cells for 4 days. The cells were then incubated overnight in low-glucose, serum-free medium followed by 24-h treatment in the presence of 2-[N-(7-nitrobenz-2-oxa-1, 3-diazol-4-yl) amino]-2-deoxy-d-glucose (2-NBDG). The fluorescence was read at excitation and emission wavelengths of 485 and 535 nm.

### 2.8. Western Blot Analysis

C2C12-derived myotubes were harvested with lysis buffer (50 mM Tris HCl, 1% Triton X-100, 0.5% sodium deoxycholate, 150 mM NaCl, 1 mM EDTA, 1 mM PMSF, 1 mM sodium orthovanadate, 1 mM NaF and 0.2% protease inhibitor cocktail; pH 7.2). Proteins (40 *μ*g) were subjected to sodium dodecyl sulfate polyacrylamide gel electrophoresis (SDS-PAGE) followed by transfer onto a nitrocellulose membrane. Protein phosphorylation or expression was detected by Western blot analysis using primary antibodies specific for phospho-AMP protein kinase (AMPK) and *β*-actin (Cell Signaling Technology, Beverly, MA).

### 2.9. Peroxisome Proliferator-Activated Receptor (PPAR)-*γ* Transcriptional Activity Assay

Plasmids for PPAR-*γ* or retinoid X receptor (RXR)-*α*, as well as luciferase reporter vectors containing PPAR-response elements (PPREs), were the generous gift of Dr. Jae Bum Kim (Seoul National University, South Korea). For the PPAR-*γ* assay, human embryonic kidney (HEK) 293 cells were cotransfected with PPAR-*γ* or RXR-*α* plasmids and PPAR-PPRE luciferase reporter vectors by using a transfection reagent (Qiagen, Valencia, CA) for 18 h. Cells were then treated with fermented *Morinda citrifolia* ethanol extract (FMCE) for 24 h, and PPAR-*γ* activity was measured via a Luciferase Assay System (Promega, Madison, WI). 

### 2.10. Statistical Analysis

Numerical data are expressed as means ± the standard deviation using SAS 9.1software. The significance of differences between experimental conditions was analyzed using one-way analysis of variance (ANOVA) followed by Duncan's multiple range test. Values of *P* < 0.05 were considered significant. 

## 3. Results

### 3.1. Blood Glucose and HbA1c Levels

**Figure 2:**
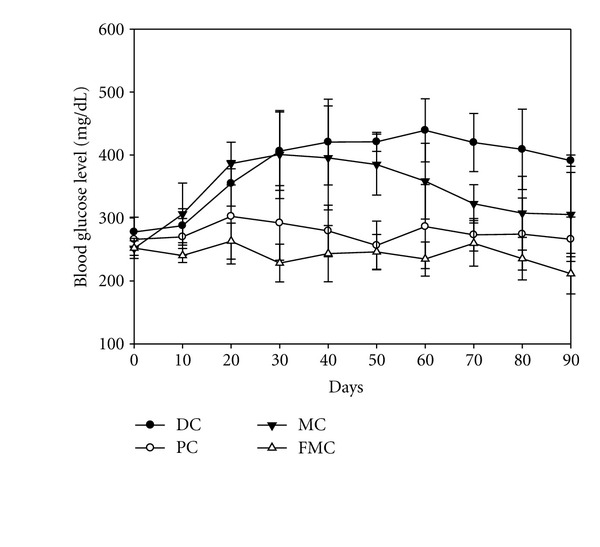
Effect of FMC on blood glucose levels in KK-Ay mice during the 90 days of experimentation. DC: diabetic control, PC: positive control (banaba), MC: *Morinda citrifolia*, FMC: fermented *Morinda citrifolia.* Each data point represents the mean ± SD (*n* = 7).

Figure 2 shows nonfasting blood glucose levels measured every ten days over the experimental period of 90 days. Blood glucose levels were remarkably increased in the DC group compared with the PC (banaba fed) group after 20 days, and were maintained at more than 400 mg/dL throughout the remaining 70 days of the experiment. Blood glucose levels in the MC group also increased to the same extent as that observed for the DC group during the first 40 days of dietary evaluation. After 40 days, the levels gradually decreased to about 300 mg/dL at 70 days and were then maintained at this level for the next 20 days. In contrast, blood glucose levels did not increase in the FMC group relative to the PC group, and the levels were maintained at 211.60–252.20 mg/dL during the experimental period. The blood glucose levels in the FMC group were actually lowerthan those in the PC group. 

**Figure 3:**
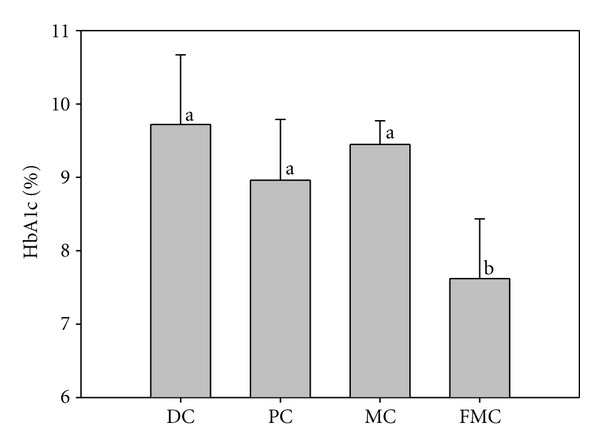
Effect of FMC on HbA1c levels (%) in KK-Ay mice after 90 days. DC: diabetic control, PC: positive control (banaba), MC: *Morinda citrifolia*, FMC: fermented *Morinda citrifolia.* Each bar represents the mean ± SD (*n* = 7). Bars with different letters represent values that are significantly different from each other based on one-way ANOVA and the Duncan's multiple range test (*P* < 0.05).

Figure 3 shows HbA1c levels at 90 days. The mean Hb1Ac level in the FMC group was 7.62%, which was significantly lower than the Hb1Ac levels in the other three groups. The corresponding values were 9.45% in the DC group, 8.96% in the PC group, and 8.96 in the MC group. 

### 3.2. Serum Glucose and Insulin Levels

**Figure 4:**
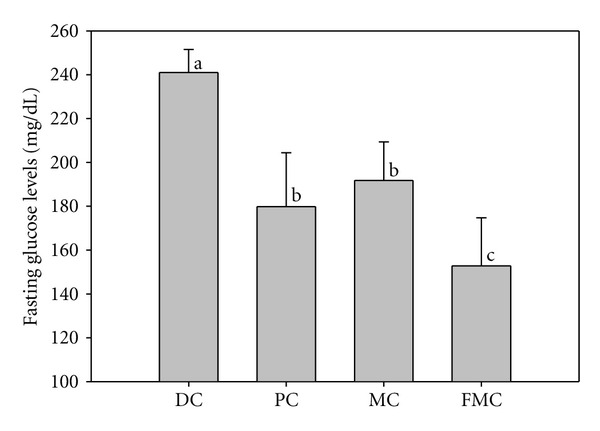
Effect of FMC on serum fasting glucose levels in KK-Ay mice after 90 days. DC: diabetic control, PC: positive control (banaba), MC: *Morinda citrifolia*, FMC: fermented *Morinda citrifolia.* Each bar represents the mean ± SD (*n* = 7). Bars with different letters represent values that are significantly different from each other based on one-way ANOVA and the Duncan's multiple range test (*P* < 0.05).

**Figure 5:**
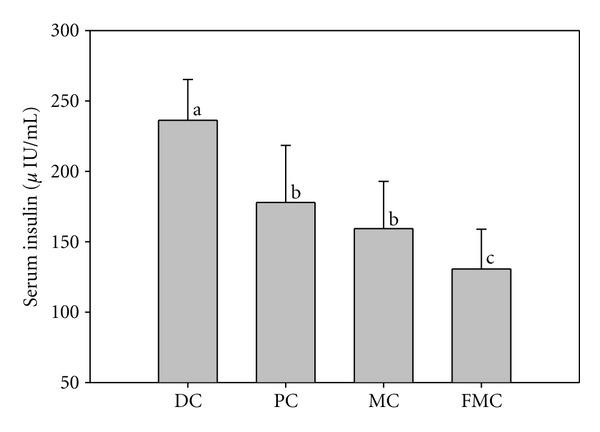
Effect of FMC on serum insulin levels in KK-Ay mice after 90 days. DC: diabetic control, PC: positive control (banaba), MC: *Morinda citrifolia*, FMC: fermented *Morinda citrifolia.* Each bar represents the mean ± SD (*n* = 7). Bars with different letters represent values that are significantly different from each other based on one-way ANOVA and the Duncan's multiple range test (*P* < 0.05).

**Figure 6:**
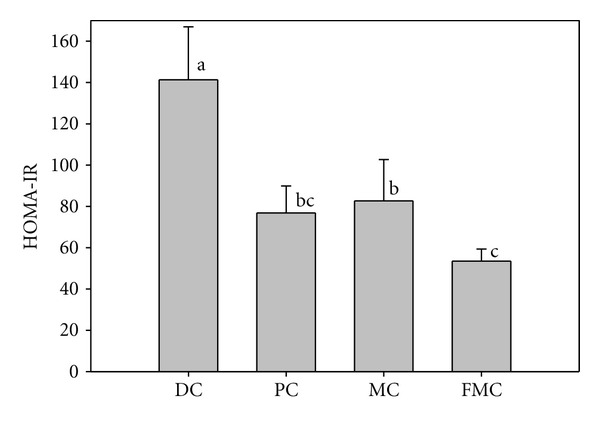
Effect of FMC on the insulin resistance index HOMA score (HOMA-IR) after 90 days. DC: diabetic control, PC: positive control (banaba), MC: *Morinda citrifolia*, FMC: fermented *Morinda citrifolia.* Each bar represents the mean ± SD (*n* = 7). Bars with different letters represent values that are significantly different from each other based on one-way ANOVA and the Duncan's multiple range test (*P* < 0.05).

Serum glucose (Figure 4) and insulin (Figure 5) levels were significantly decreased in the PC, MC, and FMC groups at 90 days relative to the DC group. The FMC group in particular showed decreased serum glucose and insulin levels by about 36% and 45%, respectively (Figures 4 and 5). The HOMA-IR index for the PC group, MC group, and FMC group were also all significantly decreased compared with the control. This result was especially evident in the FMC group (Figure 6).

### 3.3. Serum Lipid Profiles

Triglyceride levels were significantly decreased in the MC (169.00 mg/dL) and FMC (148.40 mg/dL) groups compared with the DC (236.60 mg/dL) and PC (236.60 mg/dL) groups (Table 1). Triglyceride levels in the MC and FMC groups decreased by about 29% and 37%, respectively, relative to the DC group. No significant differences were observed in total cholesterol between the groups; however, HDL-cholesterol levels in the MC and FMC groups were significantly higher than those observed in the DC group, whereas LDL-cholesterol levels in the FMC group were significantly lower than those observed in the other three groups (Table 1).

### 3.4. Glucose Uptake

**Figure 7:**
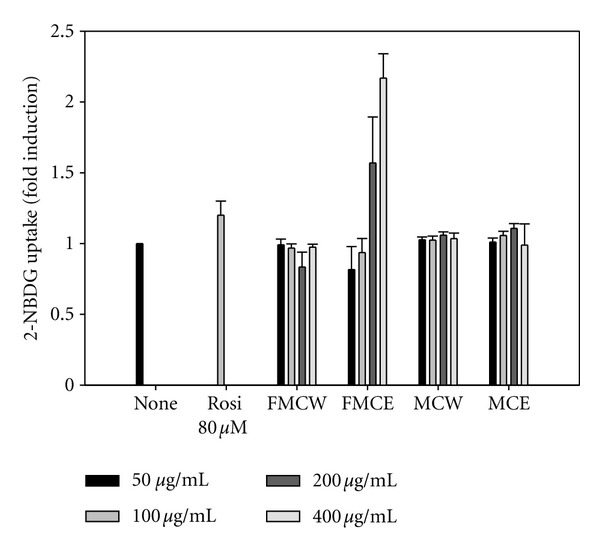
Effect of FMC extract on glucose uptake in C2C12 cells. FMCW: fermented *Morinda citrifolia *water extract, FMCE: fermented *Morinda citrifolia *70% ethanol extract, MCW: *Morinda citrifolia *water extract, MCE: *Morinda citrifolia* 70% ethanol extract. Rosi: rosiglitazone. Each bar represents the mean ± SD (*n* = 7).

To establish the potential antidiabetic mechanisms of FMC, we evaluated the effect of an ethanol extract of FMC (FMCE) on 2-NBDG uptake. FMCE significantly stimulated the uptake of 2-NBDG into C2C12-derived myotubes in a dose-dependent manner (Figure 7). When the cells were treated with FMCE at concentrations of 200 and 400 *μ*g/mL, FMCE stimulated 2-NBDG uptake by 1.6-fold and 2.2-fold, respectively, compared with untreated control cells. On the other hand, FMCW (FMC water extract), MCW (MC water extract), and MCE (MC ethanol extract) did not stimulate 2-NBDG uptake. Rosiglitazone was employed as a control because it has been shown to stimulate glucose uptake; however, it was less effective than FMCE in this effect assay.

### 3.5. PPAR-*γ* Agonist Activity

**Figure 8:**
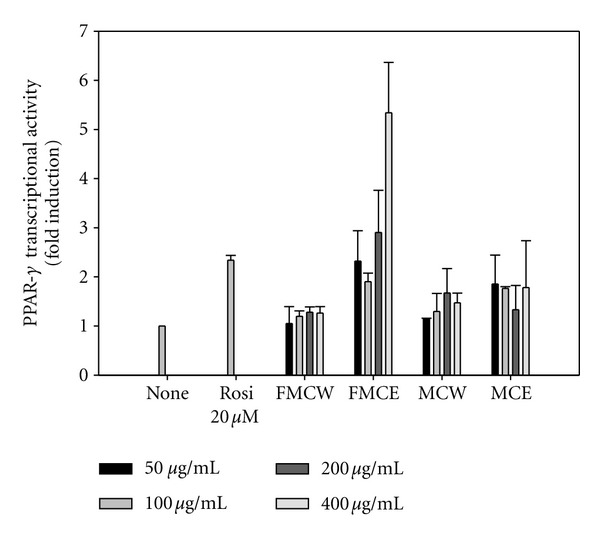
PPAR-*γ* agonist activity of FMCE compared with FMCW, MCE and MCW. FMCW: fermented *Morinda citrifolia *water extract, FMCE: fermented *Morinda citrifolia *70% ethanol extract, MCW: *Morinda citrifolia *water extract, MCE: *Morinda citrifolia* 70% ethanol extract. Rosi: rosiglitazone. Each bar represents the mean ± SD.

As shown in Figure 8, FMCE stimulated the transcriptional activity of PPAR-*γ* in a dose-dependent manner as assessed by using a luciferase assay, but FMCW, MCW and MCE did not. The PPAR-*γ* agonist activity of FMCE was comparable or even better than that of rosiglitazone, a known commercial PPAR-*γ* agonist. 

### 3.6. Phosphorylation of AMPK

**Figure 9:**
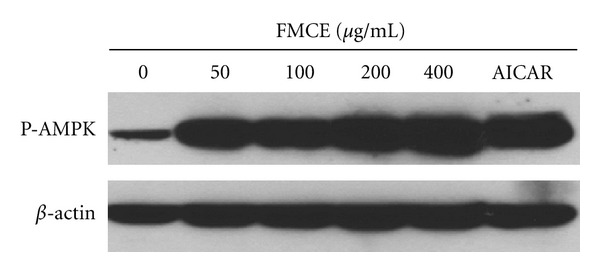
Effect of FMCE on the stimulation of AMPK activity as assessed by levels of P-AMPK. *β*-actin was used as a loading control. FMCE: fermented *Morinda citrifolia *70% ethanol extract.

To examine whether FMCE can increase the phosphorylation of AMPK, western blotting analysis was performed. As shown in Figure 9, FMCE markedly stimulated the phosphorylation of AMPK in C2C12-derived myotubes in a dose-dependent manner. In contrast, FMCW did not induce AMPK phosphorylation (data not shown). 

## 4. Discussion

The results of this study clearly indicate that the antidiabetic effects of FMC were stronger than those of the commercially available, functional health food, banaba. For example, blood glucose levels are an important indicator of diabetic status, and an FMC-based diet was more effective in reducing blood (Figure 2) and serum (Figure 4) glucose levels in KK-Ay mice than a normal diet. As reported in previous findings [13], MC also gradually reduced blood glucose levels after 40 days of experimentation; however, FMC was far more efficient in this regard compared with MC. It can be presumed that the antidiabetic activity of *M. citrifolia* was markedly improved through solid substrate fermentation, a bioconversion technique employed to increase the function of natural products. This result would be in agreement with recent studies [15, 16] demonstrating that the pharmacological effects of medicinal plants are increased through solid substrate fermentation. 

Glycosylated hemoglobin is considered to be the gold-standard indicator for the accurate and reliable measurement of fasting glucose. Fasting glucose levels are strongly associated with the extent of ambient glycemia during the 3-month period prior to fasting, as well as with the degree of protein glycation. Paralleling the results for blood glucose levels, the amount of glycosylated hemoglobin in the FMC group was lower than that in the other test groups (Figure 3). Hence, FMC can assist in the maintenance of low blood glucose levels and the control of blood glucose over the long term. 

This hypoglycemic action of FMC was associated with a decrease in both insulin content (Figure 5) and insulin resistance (Figure 6). In the control group, insulin content in addition to blood glucose levels was high relative to the other groups, particularly the FMC group. This is indicative of hyperinsulinemia and hyperglycemia caused by insulin resistance in DC mice. Notably, the HOMA-IR index (an index of insulin resistance) was significantly decreased in the FMC group compared with the DC group. The HOMA-IR index provides a simple method to measure insulin sensitivity, and its use is widespread in both clinical and animal studies [17]. 

Insulin resistance, or the failure of normal insulin-induced actions in major tissues including the liver, fat, and skeletal muscle, plays a key role in the pathophysiology of T2DM. A primary feature of insulin resistance is the reduction of glucose uptake into muscle cells. We therefore used C2C12 skeletal muscle cells to evaluate whether FMC increases glucose uptake. Our results indicate that FMCE stimulated uptake of 2-NBDG into terminally differentiated C2C12-derived myotubes in a dose-dependent manner (Figure 7). Thus, stimulation of glucose uptake may be one mechanism by which FMC improved insulin resistance in KK-Ay mice. MCW and MCE also increased glucose uptake into muscle cells; however, the effects of these extracts were lower than the effect of FMCE. These results indicate that the glucose disposal rate mediated by MC in muscle cells was increased by solid substrate fermentation.

Recently, many researchers have reported that various plant-derived flavonoids, anthraquinones, and terpenes stimulate glucose uptake in cells [18–20], reducing insulin resistance. In addition, insulin resistance has been reported to be associated with inflammation and oxidative stress [21]. Consequently, compounds such as flavonoids and lignans, which possess powerful antiinflammatory and antioxidant properties, may be useful in the control of T2DM. It has been reported that *M. citrifolia* contains numerous anthraquinones, flavonol glycosides and terpenoids, which possess antioxidant, antiinflammatory and antidyslipidemic effects [22–24]. Therefore, these compounds were likely increased in FMC by fermentation of MC, leading to at least some of the antidiabetic actions of FMC. However, further detailed study will be required to identify antidiabetic compounds in FMC. 

AMPK functions as a sensor of cellular energy that can be activated by glucose deprivation and high AMP-to-ATP ratios [25]. There are indications that the activation of this enzyme is beneficial for the treatment and prevention of T2DM and metabolic syndrome [26, 27], because its phosphorylation leads to increased glucose uptake and lipid oxidation in muscle cells [28, 29]. To investigate whether the effect of FMC on glucose uptake was mediated through AMPK activation, C2C12-derived myotubes were treated with FCME at various concentrations. As shown in Figure 9, FMCE dose-dependently increased the phosphorylation of AMPK. The actions of FMCE were similar to those of AICAR (5-amino-4-imidazolecarboxamide riboside), an AMPK-specific activator. These data are in agreement with recent reports by Lee et al. [30] and Benhaddou-Andaloussi et al. [31], which demonstrated an increase in cellular glucose uptake as a result of AMPK activation. 

PPAR-*γ* is a transcription factor that regulates gene expression in the liver, adipose tissue, vascular endothelium, and muscle [32]. PPAR-*γ* can improve insulin sensitivity and glucose tolerance via the regulation of lipid storage, glucose homeostasis, and adipokine production [33, 34]. We found that FMCE functioned as a PPAR-*γ* agonist to increase PPAR-*γ*-dependent luciferase activity *in vitro*. This suggests that FMC acts on PPAR-*γ* as well as AMPK to augment glucose uptake. FMCE (400 *μ*g/mL) showed enhanced PPAR-*γ* agonist relative to the TZD drug rosiglitazone (20 *μ*M). TZDs are widely used as PPAR-*γ* agonists in the treatment of diabetes, and TZD-mediated activation of PPAR-*γ* results in marked improvement in blood glucose levels and insulin sensitivity [35]. PPAR-*γ* agonists also significantly decrease total cholesterol and triglyceride in type 2 diabetic patients and model animals [36, 37]. The current results indicate that FMC can now be considered a new type of PPAR-*γ* agonists. 

Hypertriglyceridemia and hypercholesterolemia are central components of diabetic dyslipidemia, occurring in 20–60% of all diabetic patients. Dyslipidemia characterized by high plasma levels of total cholesterol and triglycerides, in addition to low plasma levels of HDL cholesterol, is among the many risk factors for atherosclerotic cardiovascular disease. The latter is in turn a major cause of morbidity in T2DM patients [38]. In this study, dietary supplementation with FMC caused a significant decrease in serum triglycerides and LDL-cholesterol and a concomitant increase in HDL-cholesterol. This improvement of the lipid profile may be attributed to the PPAR-*γ* agonist activity of FMC, at least in part. 

In summary, the decrease in blood glucose level mediated by FMC was associated with a significant reduction in insulin resistance, as revealed by the plasma insulin content and blood glucose levels *in vivo* in a KK-Ay mouse model of T2DM. This enhanced insulin sensitivity was related to elevated glucose disposal rates in C2C12 myotubes via the activation of PPAR-*γ* and AMPK. In addition, FMC ameliorated dyslipidemia in KK-Ay mice, most likely by stimulating PPAR-*γ*. These pleiotropic antidiabetic actions provide strong support for the use of FMC for the treatment of diabetes. However, high-quality clinical studies are essential to determine the optimal conditions for complementary or alternative treatment in diabetic patients.

## Figures and Tables

**Table 1:** Effect of FMC on total serum cholesterol, HDL-cholesterol, LDL-cholesterol and triglyceride levels (mg/dL) in KK-Ay mice after 90 days.

	Total cholesterol	HDL-cholesterol	LDL- Cholesterol	Triglycerides
Diabetic control (DC)	141.25 ± 16.69	116.00 ± 6.12^b^	9.60 ± 2.06^a^	235.60 ± 46.73^a^
Positive control (PC)	144.80 ± 12.54	119.33 ± 9.48^ab^	9.75 ± 3.90^a^	221.50 ± 31.05^ab^
MC	137.80 ± 13.53	133.40 ± 11.20^a^	9.40 ± 2.15^a^	169.00 ± 17.02^ab^
FMC	137.00 ± 12.47	130.40 ± 10.13^a^	7.80 ± 1.17^b^	148.40 ± 17.07^b^

Each value represents the mean ± SD (*n* = 7).

^a-b^Means with different superscripts in the same column are significantly different from each other (*P* < 0.05, Duncan's multiple range test).

## References

[1] Alberti KGMM, Zimmet PZ (1998). Definition, diagnosis and classification of diabetes mellitus and its complications. Part 1sdiagnosis and classification of diabetes mellitus. Provisional report of a WHO consultation. *Diabetic Medicine*.

[2] American Diabetes Association (2004). Diagnosis and classification of diabetes mellitus. *Diabetes Care*.

[3] Lin Y, Sun Z (2010). Current views on type 2 diabetes. *Journal of Endocrinology*.

[4] Chattopadhyay RR (1999). A comparative evaluation of some blood sugar lowering agents of plant origin. *Journal of Ethnopharmacology*.

[5] Miura T, Ueda N, Yamada K (2006). Antidiabetic effects of corosolic acid in KK-Ay diabetic mice. *Biological and Pharmaceutical Bulletin*.

[6] Baskaran K, Ahamath BK, Shanmugasundaram KR, Shanmugasundaram ERB (1990). Antidiabetic effect of a leaf extract from *Gymnema sylvestre* in non-insulin-dependent diabetes mellitus patients. *Journal of Ethnopharmacology*.

[7] Iizuka Y, Sakurai E, Tanaka Y (2001). Antidiabetic effect of folium mori in GK rats. *Yakugaku Zasshi*.

[8] Oh WK, Lee CH, Lee MS (2005). Antidiabetic effects of extracts from *Psidium guajava*. *Journal of Ethnopharmacology*.

[9] Hirazumi A, Furusawa E, Chou SC, Hokama Y (1996). Immunomodulation contributes to the anticancer activity of *Morinda citrifolia* (Noni) fruit juice. *Proceedings of the Western Pharmacology Society*.

[10] Nayak BS, Sandiford S, Maxwell A (2009). Evaluation of the wound-healing activity of ethanolic extract of *Morinda citrifolia* L. leaf. *Evidence-Based Complementary and Alternative Medicine*.

[11] Wang MY, Su C (2001). Cancer preventive effect of *Morinda citrifolia* (Noni). *Annals of the New York Academy of Sciences*.

[12] Singh SK, Rai PK, Jaiswal D, Watal G (2008). Evidence-based critical evaluation of glycemic potential of Cynodon dactylon. *Evidence-Based Complementary and Alternative Medicine*.

[13] Nayak BS, Marshall JR, Isitor G, Adogwa A (2011). Hypoglycemic and hepatoprotective activity of fermented fruit juice of *Morinda citrifolia* (noni) in diabetic rats. *Evidence-Based Complementary and Alternative Medicine*.

[14] Srinivasan K, Ramarao P (2007). Animal models in type 2 diabetes research: an overview. *Indian Journal of Medical Research*.

[15] Lim SI, Lee BY (2010). Anti-diabetic effect of material fermented using rice bran and soybean as the main ingredient by Bacillus sp. *Journal of Korean Society Application Biological Chemistry*.

[16] Lim SI, Cho CW, Choi UK, Kim YC (2010). Antioxidant activity and ginsenoside pattern of fermented white ginseng. *Journal of Ginseng Research*.

[17] Matthews DR, Hosker JP, Rudenski AS (1985). Homeostasis model assessment: insulin resistance and *β*-cell function from fasting plasma glucose and insulin concentrations in man. *Diabetologia*.

[18] Zhang WY, Lee JJ, Kim IS, Kim Y, Park JS, Myung CS (2010). 7-0-methylaromadendrin stimulates glucose uptake and improves insulin resistance in vitro. *Biological and Pharmaceutical Bulletin*.

[19] Saito T, Abe D, Sekiya K (2008). Sakuranetin induces adipogenesis of 3T3-L1 cells through enhanced expression of PPAR*γ*2. *Biochemical and Biophysical Research Communications*.

[20] Vessal M, Hemmati M, Vasei M (2003). Antidiabetic effects of quercetin in streptozocin-induced diabetic rats. *Comparative Biochemistry and Physiology C*.

[21] Shoelson SE, Lee J, Goldfine AB (2006). Inflammation and insulin resistance. *Journal of Clinical Investigation*.

[22] Okusada K, Nakamoto K, Nishida M (2011). The antinociceptive and anti-inflammatory action of the CHCl 3-soluble phase and its main active component, damnacanthal, isolated from the root of *Morinda citrifolia*. *Biological and Pharmaceutical Bulletin*.

[23] Su BN, Pawlus AD, Jung HA, Keller WJ, McLaughlin JL, Kinghorn AD (2005). Chemical constituents of the fruits of *Morinda citrifolia* (Noni) and their antioxidant activity. *Journal of Natural Products*.

[24] Pawlus AD, Su BN, Keller WJ, Kinghorn AD (2005). An anthraquinone with potent quinone reductase-inducing activity and other constituents of the fruits of *Morinda citrifolia* (Noni). *Journal of Natural Products*.

[25] Hardie DG (2003). Minireview: the AMP-activated protein kinase cascade: the key sensor of cellular energy status. *Endocrinology*.

[26] Fryer LGD, Parbu-Patel A, Carling D (2002). The anti-diabetic drugs rosiglitazone and metformin stimulate AMP-activated protein kinase through distinct signaling pathways. *Journal of Biological Chemistry*.

[27] Zhou G, Myers R, Li Y (2001). Role of AMP-activated protein kinase in mechanism of metformin action. *Journal of Clinical Investigation*.

[28] Zang M, Zuccollo A, Hou X (2004). AMP-activated protein kinase is required for the lipid-lowering effect of metformin in insulin-resistant human HepG2 cells. *Journal of Biological Chemistry*.

[29] Cuthbertson DJ, Babraj JA, Mustard KJW (2007). 5-Aminoimidazole-4-carboxamide 1-*β*-D-ribofuranoside acutely stimulates skeletal muscle 2-deoxyglucose uptake in healthy men. *Diabetes*.

[30] Lee MS, Hwang JT, Kim SH (2010). Ginsenoside Rc, an active component of Panax ginseng, stimulates glucose uptake in C2C12 myotubes through an AMPK-dependent mechanism. *Journal of Ethnopharmacology*.

[31] Benhaddou-Andaloussi A, Martineau LC, Vallerand D (2010). Multiple molecular targets underlie the antidiabetic effect of Nigella sativa seed extract in skeletal muscle, adipocyte and liver cells. *Diabetes, Obesity and Metabolism*.

[32] Leiter LA (2006). Can thiazolidinediones delay disease progression in type 2 diabetes?. *Current Medical Research and Opinion*.

[33] Spiegelman BM (1998). PPAR-*γ*: adipogenic regulator and thiazolidinedione receptor. *Diabetes*.

[34] Cho MC, Lee K, Paik SG, Yoon DY (2008). Peroxisome proliferators-activated receptor (PPAR) modulators and metabolic disorders. *PPAR Research*.

[35] Girard J (2001). Mechanisms of action of thiazolidinediones. *Diabetes and Metabolism*.

[36] Kwon DY, Kim DS, Yang HJ, Park S (2011). The lignan-rich fractions of Fructus Schisandrae improve insulin sensitivity via the PPAR-*γ* pathways in *in vitro* and *in vivo* studies. *Journal of Ethnopharmacology*.

[37] Nagashima K, Lopez C, Donovan D (2005). Effects of the PPAR*γ* agonist pioglitazone on lipoprotein metabolism in patients with type 2 diabetes mellitus. *Journal of Clinical Investigation*.

[38] Freed MI, Ratner R, Marcovina SM (2002). Effects of rosiglitazone alone and in combination with atorvastatin on the metabolic abnormalities in type 2 diabetes mellitus. *American Journal of Cardiology*.

